# Electrochemical fluorescence modulation enables simultaneous multicolour imaging

**DOI:** 10.1038/s41566-025-01672-7

**Published:** 2025-05-02

**Authors:** Ying Yang, Yuanqing Ma, Alexander Macmillan, Richard Tilley, J. Justin Gooding

**Affiliations:** 1 School of Chemistry and Australian Centre for NanoMedicine, University of New South Wales, Sydney, New South Wales Australia; 2 Katharina Gaus Light Microscopy Facility, University of New South Wales, Kensington, New South Wales Australia

**Keywords:** Fluorescence imaging, Super-resolution microscopy, Fluorescent probes

## Abstract

Multicolour fluorescence imaging is crucial to simultaneously visualize multiple targets in cells, enabling the study of complicated cellular processes. Common multicolour methods rely on using fluorophores with sufficiently different spectral or lifetime characteristics. Here we present a new multicolour imaging strategy on a standard fluorescence microscope, where up to four fluorophores with high spectral overlap can be resolved using a single-colour optical configuration. We find that under electrochemical modulation, the fluorophores are regulated between the bright and dim states, with each displaying a distinct fluorescence response pattern. These unique fluorescence potential profiles enable the effective separation of different fluorophores through linear unmixing. We also demonstrate that electrochemical fluorescence switching is readily applicable to four-colour STED imaging. With no modification to the optical setups and easy adaptation to different microscopes, we anticipate that colour unmixing based on electrochemical fluorescence switching will provide an easily accessible multicolour imaging pathway for discoveries in diverse fields.

## Abstract (web summary)

Electrochemical modulation of fluorophores enables regulating their emission states, facilitating spectral unmixing of up to four fluorophores with similar spectral characteristics. This method is readily applicable to multicolour STED imaging, effectively expanding a single imaging channel to four channels.

## Main

Fluorescence microscopy is widely used for visualizing biological specimens with high specificity and sensitivity^1^. Being able to visualize multiple molecular species within cells is of the utmost importance for understanding complex cellular processes, which makes multiplexing microscopy a valuable approach. Currently, the separation of fluorescence signals is commonly achieved using glass-coated beamsplitters in widefield microscopes or acousto-optical beamsplitters in laser scanning confocal microscopes^1^. Using this method, the emission spectrum of the labelled fluorophores must differ substantially to avoid spectral cross-talk, the number of resolvable colours is, thus, limited by the available excitation lasers and the corresponding emission filter configurations. To expand the range of colour options, efforts have been made to develop fluorescent probes that extend beyond the visible region^2–4^.

Progress in optical instruments has granted the opportunity for other multicolour imaging methods. With multichannel spectral detectors, fluorophores with substantial emission spectral overlap can be separated using a linear unmixing approach^5,6^. A similar concept has been used for excitation spectral unmixing, where the excitation wavelength from a white light source was scanned through an acousto-optic tunable filter, as single-emission detection remains unchanged^7,8^. In some cases, spectral unmixing alone is insufficient to resolve fluorophores that have almost identical spectroscopic characteristics. Fluorescence lifetime would become another option to discriminate fluorophores when they have similar emission spectra but sufficiently different lifetimes^9,10^. In addition to fluorescence spectra and lifetime, other photophysical properties have been explored for the separation of mixed fluorophores. For instance, different fluorophores exhibit varying levels of photostability, leading to the possibility of achieving separation based on their distinct characteristic photobleaching rates^11,12^. Another example is the unmixing of photoswitchable fluorophores based on their variation in modulation depth^13,14^.

Recently, we discovered that the fluorescence intensity of some organic dyes or fluorescent proteins are sensitive to an external electrochemical potential^15,16^. Changes in fluorescence with potential have also been shown for various families of organic fluorophores, such as rhodamine derivatives, perylene diimides and tetrazine derivatives^17–19^. These discoveries allude to the possibility for fluorophore unmixing based on their characteristic electrochemical fluorescence modulation. Specifically, we exploit differences in fluorescence intensity change in response to a varying potential to separate fluorophores that are otherwise spectrally similar. The idea presented here is to implement a simple multicolour fluorescence imaging approach without hardware modification of the microscopy system. On the basis of electrochemical fluorescence modulation, we demonstrated that fluorophore unmixing could be realized on a standard fluorescence microscope or a super-resolution stimulated emission depletion (STED) microscope, expanding a single imaging channel into up to four channels, makes multicolour imaging much simpler and more affordable.

## Results and discussion

### Linear unmixing of fluorophores via electrochemical spectra

**Fig. 1:**
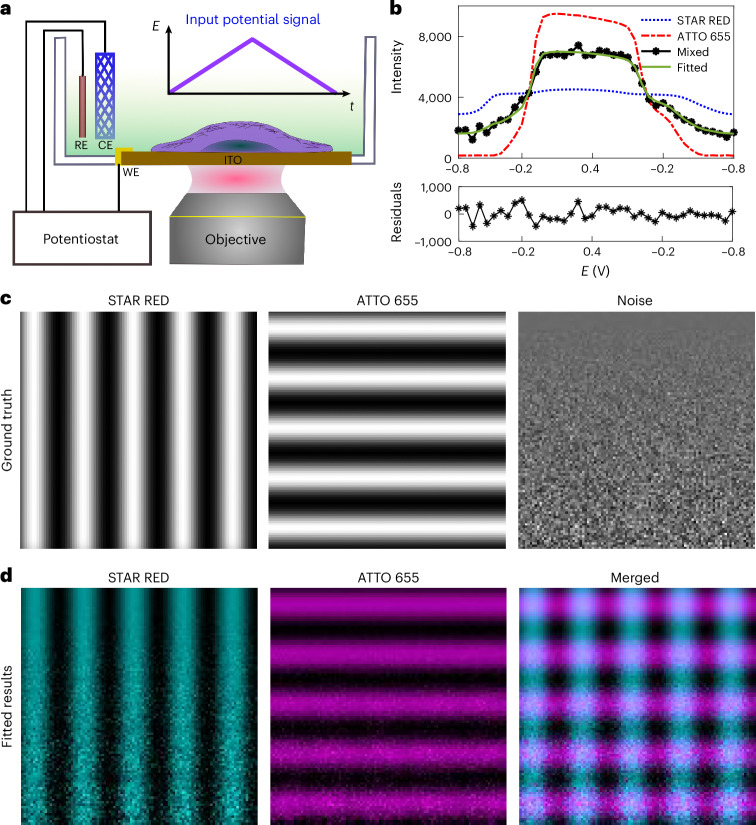
Working principle and linear unmixing of two fluorophores using simulation. **a**, Schematic of the microscope integrated with the electrochemistry setup. The inset shows the input electrochemical potential signal used to modulate the fluorophores between the bright and dim states. A typical three-electrode system is utilized, which includes a transparent ITO-coated glass coverslip as the working electrode (WE), a Ag/AgCl wire in 3 M KCl as the reference electrode (RE) and a platinum mesh as the counter electrode. Each electrode is connected to a potentiostat, which applies a defined potential difference between ITO and RE. **b**, Fluorescence signals from a STAR RED (blue line) and ATTO 655 (red line) reference dyes, as well as a 1:1 mixture of the two dyes (black line), in response to the potential change. Least-squares fitting with a non-negativity constraint was applied to extract the contribution of each fluorophore from the mixture. The fitting residual is shown below. **c**, Simulation of the two reference dyes mixed in a 100 × 100-pixel^2^ image with the indicated patterns. Gaussian noise was added along the vertical axis, ranging from 0% to 50% of the signal intensity. **d**, The two dyes were successfully unmixed, revealing patterns that correspond to the ground truth. The unmixed images are colour coded and overlaid. Source data

**Extended Data Fig. 1:**
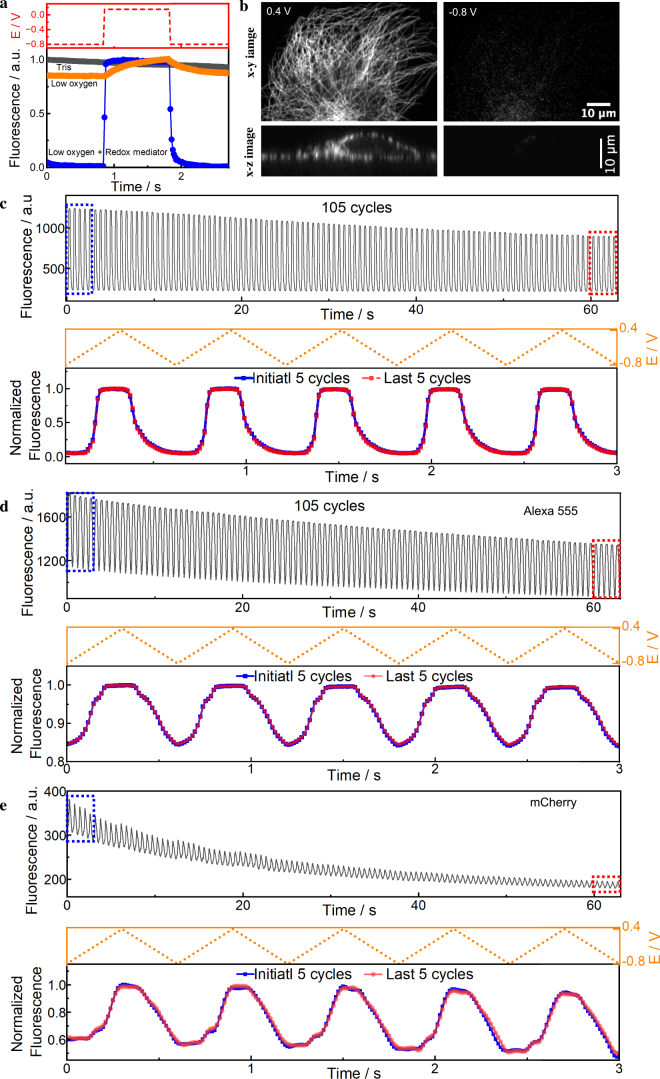
Electrochemical fluorescence modulation of ATTO 655. **a**, Fluorescence response of ATTO 655 tagged microtubules under different buffer conditions with pulsed potential between −0.8 V and 0.4 V (top panel). In Tris buffer (gray line), fluorescence remains stable, showing minimal modulation. In low-oxygen buffer (orange line), fluorescence modulation occurs slowly with very moderate change. In low-oxygen buffer with redox mediators (potassium ferricyanide and cysteamine, blue line), nearly complete on-off fluorescence modulation is achieved. **b**, Demonstration of electrochemical fluorescence modulation of ATTO 655 in the x-y and x-z planes. Both planes exhibit nearly complete on-off fluorescence modulation behaviour. The images are shown with same contrast setting. **c**, EC spectra of ATTO 655 over 105 cycles. The EC spectra from the first (blue) and last 5 cycles (red) were normalized by subtracting their background and scaling to their maximum intensities, respectively, which shows excellent overlap. **d-e**, Similarly, EC spectra for Alexa 555 and mCherry over 105 cycles demonstrates high repeatability. In c-e, the potential scan rate is 4 V s^-1^. The frame rate is 11 ms. Source data

**Extended Data Fig. 2:**
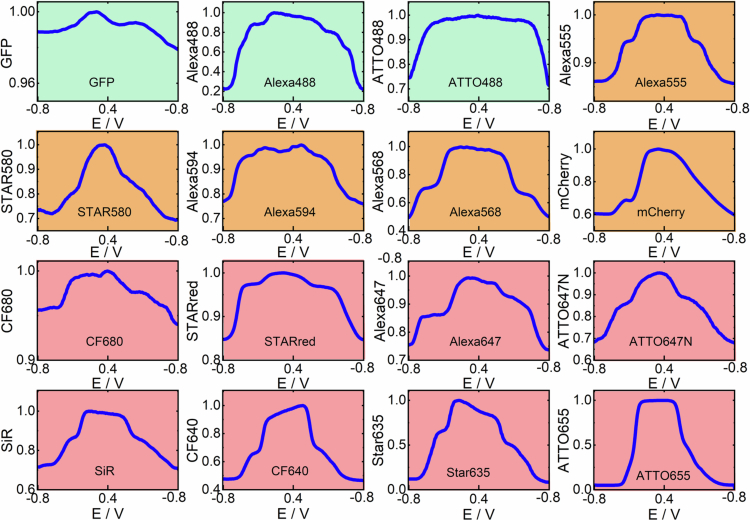
A list of EC spectra for various fluorophores. The image panels are highlighted in green, orange, and red, corresponding to the green, red and far-red spectral channels for the respective fluorophores. The EC spectra was collected from the immunostained cells with the given dye or the fluorescent protein transfected cell and imaged over a widefield epi fluorescence microscope. During the acquisition of the EC spectra, the electrochemical potential was scanned from −0.8 V to 0.4 V and back to −0.8 V at a scan rate of 500 mV s⁻¹. The EC spectra were normalized by subtracting their background and scaling to their maximum intensities. The frame rate as set at 20 ms. The imaging buffer is a low-oxygen buffer (glucose-glucose oxidase) containing cysteamine (100 mM) and ferricyanide (20 mM). Source data

To achieve simultaneous electrochemical control and optical imaging, we utilized an indium tin oxide (ITO)-coated glass coverslip, which functions as the imaging surface as well as the electrode (Fig. 1a)^20–22^. By connecting the ITO to a potentiostat with reference and auxiliary electrodes, the potential at the ITO surface can be precisely controlled. When the potential at the ITO scanned linearly as a function of time, the fluorescence intensity of the fluorophores would show their characteristic fluorescence modulation curves. Previous studies found that an external electrochemical potential can affect the fluorescence intensity of various organic dyes and fluorescent proteins^15,16,18,19^. However, direct electrochemical modulation is challenging for fluorophores on cellular structures, as most are too far from the electrode for efficient electron transfer. To overcome this limitation, we introduced a redox couple, cysteamine and ferricyanide, into a low-oxygen buffer to mediate the electrochemical fluorescence modulation in the entire cells, using ATTO 655 dye as an example (Supplementary Video 1 and Extended Data Fig. 1a,b). This type of electrochemical fluorescence modulation demonstrates high repeatability over extended cycles (Supplementary Video 2 and Extended Data Fig. 1c–e). In particular, this electrochemical fluorescence modulation is currently limited to fixed and permeabilized cells, as permeabilization is crucial for both antibody immunostaining and electrochemically mediated reactions within the cell. We then compared the fluorescence responses of ATTO 655 and STAR RED, both immunostained on the microtubules of cells attached to separate ITO surfaces, during a linear scan of the electrochemical potential (Fig. 1a,b). As the potential increased, both dyes showed a rise in fluorescence intensity, which subsequently decreased as the potential was scanned back negatively. However, their modulation profiles differed markedly. ATTO 655 demonstrated a sharp, nearly complete ON–OFF response, with fluorescence intensity shifting distinctly at positive and negative potentials. By contrast, STAR RED displayed a broader and more gradual response, with only a modest intensity change across the same potential range. Extended Data Fig. 2 provides additional examples of various fluorophores, each displaying a distinct fluorescence modulation curve across the applied potential range. These distinct responses suggest that each fluorophore has a unique pattern of fluorescence modulation under the same potential range. To describe this characteristic behaviour, we term the specific fluorescence response of a fluorophore as a function of the applied potential the ‘electrochemical spectrum’ (EC spectrum). The EC spectrum provides a framework for the unmixing of fluorophores based on their electrochemical fluorescence modulation properties.

Similar to colour unmixing based on the fluorescence spectra and fluorescence lifetime, we reasoned that fluorophores could be separated based on their EC spectrum. To test this hypothesis, we created a mixed dataset by combining two reference signals, namely, ATTO 655 (red line) and STAR RED (blue line), in a 1:1 ratio (mixed, black line). We added 20% Gaussian noise to the mixed dataset to incorporate the camera and background noise. To determine the exact contribution of each reference dye within the mixed sample, we applied least-squares fitting with a non-negativity constraint to solve the linear equations. The fitting yielded fractional contributions of 0.498 ± 0.004 and 0.502 ± 0.006 for the STAR RED and ATTO 655 signals, respectively, closely matching the expected 1:1 ratio. The residuals were symmetrically distributed around zero, further confirming the accuracy of the fit (Fig. 1b).

To further evaluate our unmixing algorithm in practical imaging conditions, we varied the ratio of the two reference fluorophores in a 100 × 100 pixel^2^ area, with their distribution patterns shown in Fig. 1c. Gaussian noise from 0% to 50% was added to the simulated image along the vertical direction (Fig. 1c). Using the EC spectra from Fig. 1b, we applied our unmixing algorithm to separate the signals. The results in Fig. 1d show the successful recovery of the true distribution patterns for the two fluorophores. The increased noise shows no impact on the unmixing algorithm. The results indicate the capability of the unmixing algorithm for separating the signals with low signal-to-noise ratios, similar to the principles of locked-in detection in deep tissue imaging^13^. This simulation confirms that our linear unmixing approach accurately extracts the contribution of each fluorophore within the mixed signal. The unmixing algorithm will be used for separating fluorophores in the experiments that follow.

### Six-colour imaging in three spectral channels

As an experimental demonstration of multicolour imaging by unmixing fluorophores from their EC spectra, we imaged three pairs of fluorophores in three spectral channels on a standard confocal fluorescence microscope. The emission spectra of each pair of fluorophores have extensive overlap, and lie in the green, red and far-red spectral channels, respectively. In detail, for the green channel, we genetically tagged the microtubules using enhanced green fluorescent protein (EGFP), and stained the intermediate filament vimentin with an anti-vimentin antibody tagged with Alexa 488. For the red channel, we genetically tagged the nucleus Histone 2B (H2B) protein with mCherry and immunostained the transferrin receptor using an anti-transferrin antibody tagged with Alexa 568. For the far-red channel, the actin cytoskeleton was stained using phalloidin conjugated with ATTO 655 dye, and the focal adhesion protein paxillin was labelled with anti-paxillin antibody tagged with STAR 635.

**Fig. 2:**
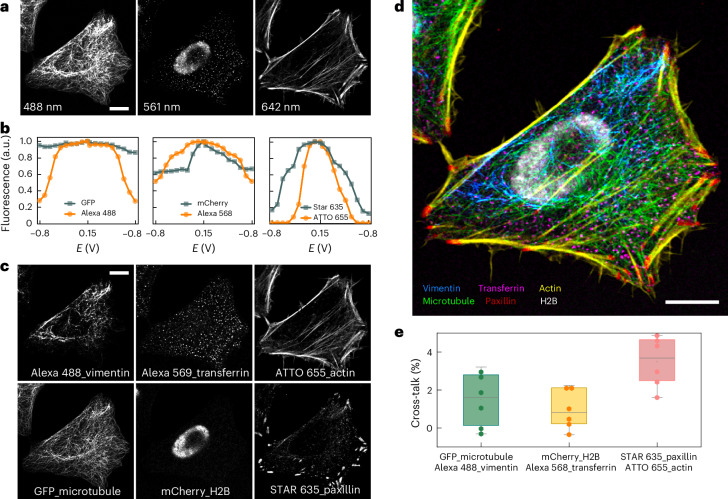
Colour unmixing of six fluorophores in three spectral channels based on electrochemical fluorescence modulation. **a**, Raw image of multitarget labelled HeLa cell before colour unmixing, where the microtubule, vimentin, cell nucleus, transferrin receptor, actin filament and paxillin are labelled with GFP, Alexa 488, mCherry, Alexa 568, ATTO 655 and Star 635, respectively. **b**, EC spectra of three pairs of fluorophores as indicated. Three time-lapse series were recorded as the electrochemical potential was scanned from −0.8 V to 0.15 V and back to −0.8 V, at a scanning rate of 500 mV s^–1^. Each time-lapse series consists of 19 frames of images at a frame rate of 200 ms. **c**,**d**, Separated (**c**) and merged (**d**) fluorescent images of a HeLa cell after colour unmixing, with different colour-coded cellular structures. Actin filaments appear in yellow, paxillin in red, microtubules in green, transferrin in magenta, vimentin filaments in blue and the nucleus in white. **e**, Cross-talk factor (*n* = 6 colour unmixing experiments on different cells), represented by the Pearson correlation coefficient, for three pairs of unmixed images across three spectral channels. The boxes extend from the 25th to 75th percentiles, with the centre lines representing the median values. The whiskers reach out to the largest and smallest values, not exceeding 1.5 times the interquartile range (IQR) from the upper- and lower-box hinges, respectively. Scale bar, 10 µm (**a**, **c** and **d**). Source data

Three time-lapse series were recorded across three spectral channels in the sequential line scanning mode as the electrochemical potential was scanned from –0.8 V to 0.15 V and back to –0.8 V in a single cycle. From the raw images across all three channels (Fig. 2a and Supplementary Video 3), distinguishing individual stained cellular structures was challenging. However, as shown in the EC spectrum for each fluorophore (Fig. 2b), the two fluorophores in each spectral channel exhibit distinct EC spectra across the potential scanning range. For example, Alexa 488 showed a more pronounced fluorescence intensity change in response to the potential change compared with EGFP. In the red channel, Alexa 568 displayed a symmetric bell-shaped EC spectrum compared with the less symmetrical response of mCherry. Similarly, in the far-red channel, ATTO 655 showed a sharper, symmetric fluorescence peak compared with the broader EC spectrum of STAR 635. These distinct EC spectra enabled a clean separation of mixed fluorophores in each channel through linear unmixing, as shown in the unmixed images (Fig. 2c) and the merged image (Fig. 2d).

To statistically evaluate the effectiveness of our unmixing technique, we quantified the cross-talk between each pair of unmixed images using the Pearson correlation coefficient, as reported in previous studies^23,24^. A Pearson correlation coefficient of 1 indicates 100% cross-talk, whereas a value of zero indicates no cross-talk. We assume the structures imaged in this study are independently and spatially distributed, and a zero or near-zero cross-correlation is anticipated between the images. Therefore, positive correlation values would suggest false-positive co-localization due to cross-talk, occurring when signals from structure A are incorrectly detected in the image of structure B. Conversely, a negative correlation would indicate incorrect unmixing, where the structure from image A is subtracted from image B. As shown in Fig. 2e, the Pearson correlation coefficient, referred to as the cross-talk factor here, is less than 0.02 for GFP-microtubule and Alexa 488-vimentin, as well as for mCherry-H2B and Alexa 568-transferrin, indicating minimal cross-talk and effective separation of the mixed fluorescent signals. In particular, an exception to the cross-talk assumption involves STAR 635-labelled paxillin and ATTO 655-tagged actin structures, unmixed from the far-red channels. These structures partially interact and function together to regulate cell movement and adhesion dynamics^25^. The Pearson correlation coefficient of 0.04 for unmixed paxillin and actin mainly reflects a biologically relevant overlap at the focal adhesions.

**Extended Data Fig. 3:**
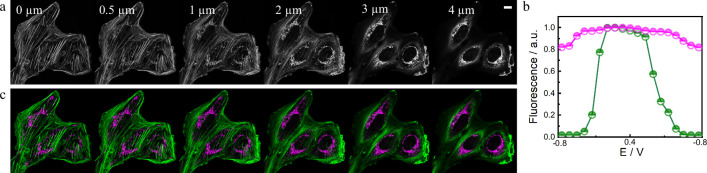
Electrochemical colour unmixing demonstration across various z-focal planes. **a**, Raw fluorescence images of U-2 OS cells with ATTO 655-labeled actin and STAR RED-labelled mitochondria at different z-distances from the ITO surface, with the indicated distances of 0, 0.5, 1, 2, 3, and 4 µm, respectively. **b**, EC-spectra for the two dyes, green for ATTO 655, magenta for STAR RED, while the electrochemical potential was scanned from −0.8 V to 0.4 V and then back to −0.8 V with a scan rate of 0.5 V/s, the frame rate is 250 ms **c**, Effective separation achieved at various z-focal planes, ATTO 655-actin was labelled in green colour, STAR RED-mitochondria was labelled in magenta. Scale bar = 10 µm. Source data

**Extended Data Fig. 4:**
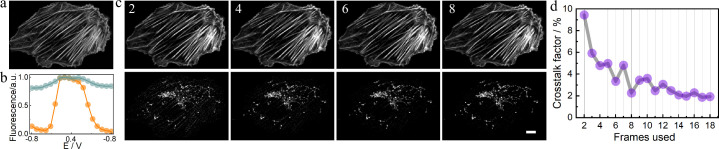
Analysis of crosstalk as function of number of frames applied for electrochemical colour unmixing. **a**, Raw images showing ATTO 655-labeled actin and STAR RED-labelled mitochondria for a U-2 OS cell. **b**, EC-spectra for STAR RED (green line) and ATTO 655 (orange line), while the electrochemical potential was scanned from −0.8 V to 0.4 V and then back to −0.8 V with a scan rate of 0.5 V/s, the frame rate is 250 ms. **c**, Unmixed images obtained using 2, 4, 6, and 8 frames, demonstrating progressive improvement in separation. **d**, Crosstalk factor as a function of the number of frames used, showing decrease as the frame number increases from 2 to 8 frames. Scale bar = 10 µm. Source data

In addition, we demonstrated that our electrochemical-based unmixing approach remains effective across various *z* distances of cells from the ITO surface (Extended Data Fig. 3 and Supplementary Video 4). Furthermore, we compared the EC spectra in both *x*–*y* and *x*–*z* imaging planes under varying potential scanning rates (Supplementary Fig. 1). A scanning rate lower than 4 V s^–^^1^ is suggested to ensure high consistency of the EC spectra in both *x*–*y* and *x*–*z* ranges. Additionally, we analysed how the number of sampling frames within an EC spectrum influences cross-talk between two fluorophores. Our findings in Extended Data Fig. 4 and Supplementary Video 5 indicate that although two frames are sufficient to separate signals, oversampling with additional frames across the EC spectrum further minimizes cross-talk, improving the unmixing accuracy. To validate our unmixing method across different imaging platforms, we tested the electrochemical-based colour unmixing on a widefield fluorescence microscope using the mCherry-H2B and Alexa 568-transferrin pair, demonstrating its effectiveness (Supplementary Fig. 2). These findings show that EC-spectrum-based colour unmixing effectively expands the number of distinguishable fluorophores that can be imaged using standard fluorescence microscopes.

### Four-colour imaging in a single channel

**Fig. 3:**
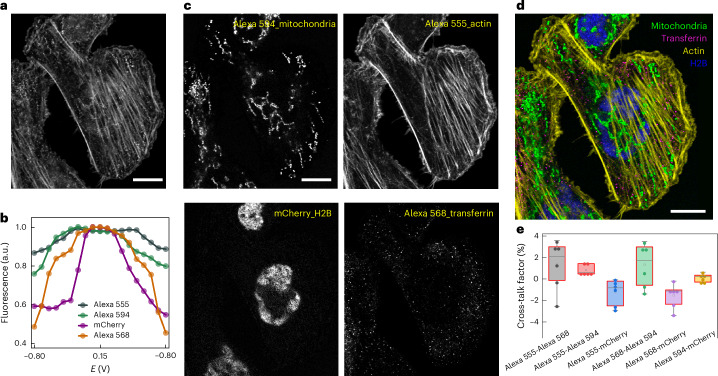
Colour unmixing of four fluorescent labels in a single spectral channel. **a**, Raw image of multitarget labelled HeLa cell before colour unmixing, where the mitochondria, actin, cell nucleus and transferrin receptor are labelled using Alexa 594, Alexa 555, mCherry and Alexa 568, respectively. **b**, Representative EC spectra for Alexa 568, mCherry, Alexa 594 and Alexa 555, as indicated. The potential was linearly scanned back and forth between −0.8 V and 0.15 V, at a scan rate of 500 mV s^–1^, and the time-lapse series consists of 19 frames of images at a frame rate of 200 ms. **c**, Unmixed images with different cellular structures as indicated. **d**, Overlaid image after colour unmixing with various structures colour-coded. Actin filaments appear in yellow; mitochondria, in green; transferrin, in magenta; and nucleus, in blue. **e**, Box plot (*n* = 6) showing the cross-talk factor for unmixed images. The boxes extend from the 25th to 75th percentiles, with the centre lines representing the median values. The whiskers reach out to the largest and smallest values, not exceeding 1.5 times the IQR from the upper- and lower-box hinges, respectively. Scale bar, 10 µm (**a**, **c** and **d**). Source data

After demonstrating the feasibility of separating two fluorescent labels in different spectral channels, we next explored the potential of resolving more fluorophores within the single channel. Specifically, we selected four red-emitting fluorophores, namely, Alexa 568, mCherry, Alexa 555 and Alexa 594, to label the cellular components: the transferrin receptor for cell surface transferrin clusters tagging, H2B for nucleus labelling, phalloidin for actin cytoskeletal staining and TOM20 for mitochondria identification, respectively. A time-lapse series of images was acquired as the electrochemical potential was scanned from −0.8 V to 0.15 V and back. Figure 3a shows the raw image, averaged from the series, where cellular structures are challenging to distinguish. From the EC spectra shown in Fig. 3b (corresponding to Supplementary Video 6), the four fluorophores exhibited varying fluorescence modulation depths between −0.8 V and 0.15 V. Alexa 568 showed the most pronounced change, followed by mCherry, Alexa 594 and Alexa 555. More importantly, there are clear differences at the inflection points, shoulder peaks and troughs on each EC spectra, making it possible to identify and separate each fluorophore through the unmixing algorithm. After performing linear unmixing, the individual unmixed images and the colour-coded overlay image (Fig. 3c,d) showed all four labelled structures as distinctly discernible. This unmixing process also effectively separated different fluorescent labels within the same pixel, resolving structures such as mitochondria and actin located beneath the cell nucleus. To assess cross-talk, we applied the Pearson correlation coefficient to analyse the unmixed image pairs (Fig. 3e). The results demonstrated minimal cross-talk, confirming the capability of our unmixing approach to separate four distinct fluorophores within a single imaging channel.

### Expanding the colour unmixing approach to STED microscopy

After demonstrating effective colour unmixing using a standard confocal fluorescence microscope, we aim to investigate the feasibility of the electrochemical-based colour unmixing method for super-resolution STED microscopy. Early implementations of multicolour STED microscopy often involved complex optical setups, with each fluorophore using its own pair of excitation and depletion lasers^26^. Achieving the precise alignment of multiple lasers poses an optical complexity. In addition, due to the first blueshifted depletion laser frequently results in direct excitation and subsequent photobleaching of the second red-shifted dye, compromising the quality of the images^27^. Recent multicolour STED implementations that use multiple excitation lasers to excite different coloured dyes but a shared depletion laser have been demonstrated^28–31^. To reduce spectral cross-talk, interleaved excitation has to be used to sequentially excite each dye over time. Due to the limited spectral overlap between the tail of the bluer spectral dyes and the depletion laser, the image resolution of the bluer dyes are often compromised^28^. In more recent developments, lifetime information has been integrated with spectral imaging for three-colour STED imaging^27,32^, where the time-correlated single-photon-counting electronic timing devices are required to reconstruct the lifetime information.

**Fig. 4:**
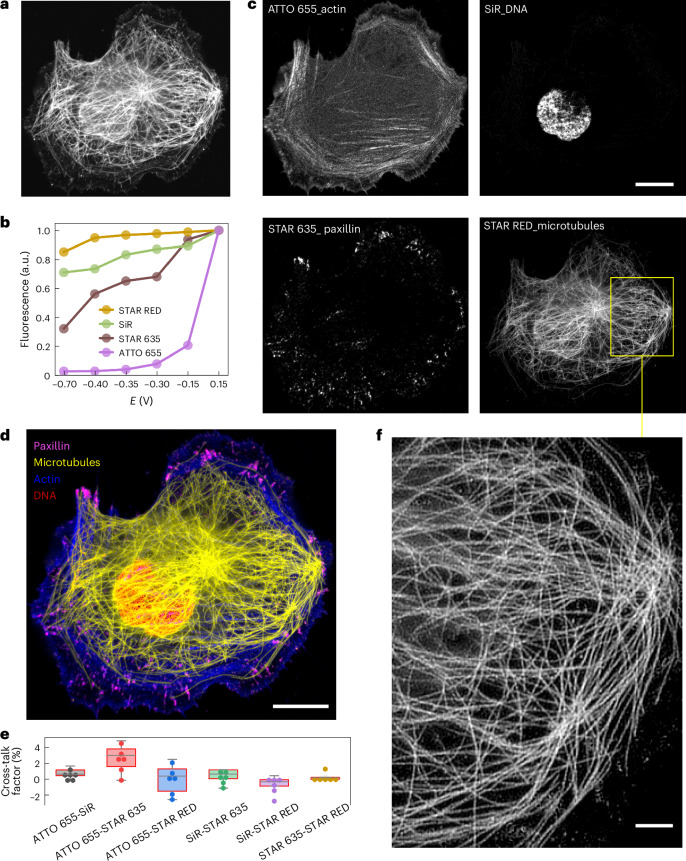
Four-colour STED imaging based on electrochemical fluorescence modulation. **a**, A U-2 OS cell was co-immunostained with STAR RED-tubulin, ATTO 655-phalloidin, SiR-DNA and STAR 635-paxillin. The raw image was generated by summing a total of six image frames. **b**, EC spectra of four dyes were measured at the respective labelled potential values; the STED images were captured at a frame rate of approximately 27 s. **c**, Electrochemical unmixed images of U-2 OS cells showing the structures for actin filaments, nucleus, paxillin and microtubules. **d**, The merged unmixed image was colour coded, with yellow representing the microtubules, magenta representing paxillin, red representing the nucleus and blue representing actin. **e**, Box plot (*n* = 6) showing the cross-talk factor for unmixed images. The boxes extend from the 25th to 75th percentiles, with the centre lines representing the median values. The whiskers reach out to the largest and smallest values, not exceeding 1.5 times the IQR from the upper- and lower-box hinges, respectively. **f**, Zoomed-in region of the unmixed microtubule structure. Scale bars, 10 µm (**c** and **d**); 2 µm (**f**). Source data

**Extended Data Fig. 5:**
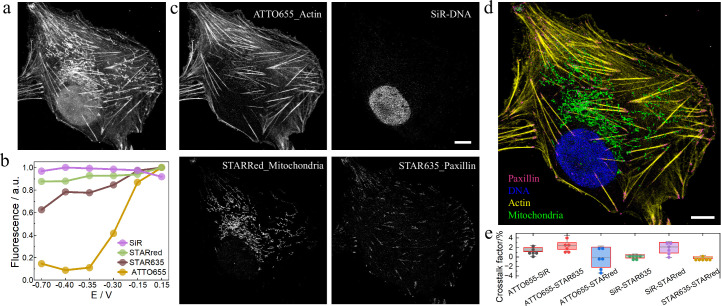
Four colour STED imaging by electrochemical colour unmixing. **a**, A U-2 OS cell was co-immunostained with STAR RED-mitochondria, ATTO 655-phalloidin, SiR-DNA, and STAR 635-paxillin. The raw image was generated by summing a total of six image frames. **b**, EC spectra of four dyes were collected at the respective labelled potential values, the STED images were captured with a frame rate of 27 s. **c**, Unmixed images of U-2 OS cells showing the structures for actin filaments, nucleus, mitochondria, and paxillin. **d**, The merged unmixed image was color-coded, with yellow representing actin, magenta representing paxillin, blue representing the nucleus, and green representing mitochondria. **e**, Box plot (n = 6 colour unmixing experiments on different cells) showing the crosstalk factor, represented by the Pearson correlation coefficient, for unmixed images. The boxes extend from the 25^th^ to 75^th^ percentiles, with the centre lines representing the median values. The whiskers reach out to the largest and smallest values, not exceeding 1.5 times the interquartile range (IQR) from the upper- and lower-box hinges, respectively. Scale bar = 10 µm. Source data

Here we explored the feasibility of performing four-colour STED imaging using a single-colour STED setup, using a single pair of excitation and depletion lasers along with a single detector. Under this optical configuration, STED images are collected at the optimal excitation and depletion wavelengths for each dye, ensuring ideal STED efficiency. Four widely used STED dyes, namely, STAR RED, STAR 635, silicon-rhodamine-based fluorophore SiR and ATTO 655, were utilized to label the microtubules, paxillin, DNA and actin within U-2 OS cells (Fig. 4a). To unmix the dyes and minimize photobleaching in multiple STED images, we collected six STED images at different electrochemical potentials (−0.7 V, −0.4 V, −0.35 V, −0.3 V, −0.15 V and 0.15 V) to construct the EC spectrum for each dye (Fig. 4b and Supplementary Video 7). This set of potentials was chosen to maximize the differences for EC spectra among the four dyes. A comparison between the confocal and STED images for the same cell shows the superior resolution under the STED imaging mode (Supplementary Fig. 3). As shown in Fig. 4c, we successfully unmixed the four dyes using our linear unmixing algorithm. Each unmixed image displayed clean and specific cellular structures, with minimal cross-talk observed among the unmixed images (Fig. 4d,e). The zoomed-in region of the unmixed microtubule structure (Fig. 4f) clearly demonstrates the super-resolution capability of STED imaging. Another set of four-colour STED images, showing actin, paxillin, nucleus and mitochondria, is shown in Extended Data Fig. 5, where the structures are also well unmixed. Overall, our electrochemical-based multicolour STED imaging approach has greatly simplified the optical configuration of multicolour STED imaging, making super-resolution multicolour STED imaging a more accessible and practical application.

## Discussion

We demonstrate a multicolour imaging technique in which the fluorescence intensity of fluorophores is electrochemically modulated and unmixed based on their unique fluorescence modulation profiles, referred to as the EC spectra. The variability in the EC spectra introduces an additional dimension for fluorescence imaging and serves as the foundation for fluorophore unmixing. In this work, we presented a set of EC spectra for various fluorophores (Extended Data Fig. 2), including rhodamine, cyanine, oxazine and fluorescent proteins, and demonstrated their application in colour unmixing. Our results suggest that when selecting fluorophores for colour unmixing, it is important to consider the chemical structures of organic dyes or chromophores. Selection should prioritize fluorophores with distinct fluorescent functional groups, such as a combination of oxazine dyes, rhodamine dyes and fluorescent proteins (Figs. 2 and 3). As a secondary criterion, fluorophores sharing the same fluorescent functional groups but with distinct terminal groups may also be considered, as they exhibit variations in EC spectral patterns, as seen with Alexa 568 and Alexa 594 (Fig. 3) or STAR RED and SiR (Fig. 4). Oversampling during image acquisition to resolve more detailed EC spectra would help improve the accuracy of colour unmixing.

In brief, our multicolour imaging approach, based on electrochemical fluorescence modulation, enables the visualization of up to four distinct cellular structures with a single-colour imaging setup, substantially expanding the number of fluorophores that can be resolved on a standard microscopy system. Using this same strategy, we also achieved four-colour super-resolution STED imaging with a single-colour STED setup without hardware modification. This approach substantially reduces the technical complexity for multicolour STED imaging, enhancing its potential for exploring subdiffraction-limit structures. The versatility of this technique, combined with its straightforward setup and compatibility with a wide range of fluorophores, makes it a powerful and affordable method for multicolour imaging on virtually any microscopy system.

## Methods

### Chemicals and materials

Commercially manufactured ITO-coated glass coverslip (06489-AB, SPI Supplies) contains a 750-nm-thick layer of ITO deposited on a 170-μm-thick glass coverslip. It produces an electric resistance of 8–12 Ω. Before placing cells on top, the ITO coverslip was plasma cleaned for 3 min and washed with 70% ethanol.

The electrochemical imaging buffer for fluorescence modulation was prepared in the following way: (1) to stock buffer A containing 50 mM Tris, add 10 mM NaCl (adjust to pH 8); (2) to buffer A, 10% glucose was added, followed by the addition of 0.5 mg ml^–1^ glucose oxidase and 40 μg ml^–1^ catalase to form an oxygen-scavenging buffer. Potassium ferricyanide and cysteamine were then added to the oxygen-scavenging buffer at final concentrations of 20 mM and 100 mM, respectively. All the chemicals are purchased from Sigma-Aldrich.

### Immunofluorescence staining

The six- or four-fluorophore labelling (Figs. 2 and 3, respectively) was achieved by using the EGFP-alpha-tubulin and H2B-mCherry stably transfected HeLa cell line (Cell Lines Service). The EC spectra for various fluorophores were collected on the COS-7 cell line (ATCC CRL-1651). The HeLa and COS-7 cells were cultured in Dulbecco’s modified Eagle’s medium fortified with 10% fetal bovine serum, penicillin and streptomycin, and incubated at 37 °C with 5% CO_2_. The four-colour STED imaging was performed on the U-2 OS cell line (Cell Lines Service). The cells were cultured in McCoys 5a medium fortified with 10% fetal bovine serum, 2.0 mM sodium pyruvate, 2.2 g l^–1^ NaHCO_3_, 1% non-essential amino acids, penicillin and streptomycin, and incubated at 37 °C with 5% CO_2_. Cells were plated in six-well plates with ITO slides in at ~10,000–20,000 cells per well on the day before fixation.

For the six-colour imaging (Fig. 2), the immunostaining procedure was performed on EGFP-alpha-tubulin and H2B-mCherry stably transfected HeLa cells as follows: before fixation, cells were incubated in transferrin conjugated with Alexa 568 (Thermo Fisher, T23365) at ~5 µg ml^−1^ for 10 min and washed with phosphate-buffered saline (PBS). Then, the cells were pre-extracted by incubating for 30 s in an extraction buffer containing 0.25% Triton, 0.1% glutaraldehyde, 80 mM 1,4-Piperazinediethanesulfonic acid (PIPES), 5 mM ethylene glycol-bis(β-aminoethyl ether)-N,N,N′,N′-tetraacetic acid (EGTA) and 2 mM MgCl_2_, and fixed for 10 min at 37 °C in a fixation buffer containing 0.25% Triton, 0.5% glutaraldehyde, 80 mM PIPES, 5 mM EGTA and 2 mM MgCl_2_. Then, the surface was quenched in 0.1% NaBH_4_ in PBS buffer at room temperature for 10 min followed by washing with PBS. Subsequently, cells were incubated for 1.5 h with a mixture of primary antibodies, which included mouse anti-paxillin antibody (Thermo Fisher, AHO0492), Alexa 488-tagged rabbit anti-vimentin antibody (Abcam, ab185030) and phalloidin ATTO 655 (18846, Sigma-Aldrich), at a concentration of 2 µg ml^−1^ in a blocking buffer (3% bovine serum albumin in PBS). Cells were then washed with PBS and incubated with STAR 635-labelled goat anti-mouse IgG secondary antibody (abberior) at a concentration of ~2.5 µg ml^−1^ for 30 min.

For the four-colour imaging in the 561-nm channel, the EGFP-alpha-tubulin and H2B-mCherry stably transfected HeLa cells were used for immunostaining. Before fixation, cells were incubated in transferrin conjugated with Alexa 568 at ~5 µg ml^−1^ for 10 min. Then, the cells were fixed and permeabilized and incubated for 1.5 h with a mixture of phalloidin Alexa 555 (A30106, Thermo Fisher) and rabbit recombinant TOM20 antibody (ab186735, Abcam). Cells were washed with PBS and incubated with Alexa 594-conjugated goat anti-rabbit secondary antibody (A11037, Thermo Fisher) at a concentration of ~2.5 µg ml^−1^ for 30 min.

For STED imaging, U-2 OS cells were used for immunostaining. After cell fixation and permeabilization, the cells were incubated with a mixture of rabbit anti-α-tubulin (ab216650, Abcam) and mouse anti-paxillin monoclonal antibody (AHO0492) at ~2 µg ml^−1^ in a blocking buffer for 1.5 h. Cells were then washed with PBS, and incubated with secondary goat anti-rabbit and goat anti-mouse IgG conjugated with STAR RED (abberior) and STAR 635, SiR-DNA (SPIROCHROME) and ATTO 655-phalloidin at a concentration of ~2.5 µg ml^−1^ for 30 min.

### Electrochemistry

All the electrochemistry measurements were carried out using an SP-200 potentiostat (Bio-Logic) in a custom chamber (Chamlide EC 22, Live Cell Instrument), containing an Ag|AgCl|3 M KCl reference electrode and a Pt-wire counter electrode, where the working electrode was the ITO-coated coverslips (8–12 Ω, 22 × 22 cm^2^, SPI Supplies). Cyclic voltammetry was used to linearly scan the potential from −0.8 V to o.15 V for colour unmixing on the confocal microscope, whereas chronoamperometry was used to apply a constant potential during the acquisition of each STED image.

### Image acquisition and analysis

The fluorescence response under a linear scanning potential (Fig. 1b, Extended Data Figs. 1 and 2 and Supplementary Figs. 1 and 2) was collected on a ZEISS Elyra 7 widefield microscope equipped with two scientific complementary metal–oxide–semiconductor (sCMOS) cameras. For excitation, one of the 488-, 561- and 642-nm lasers was reflected from a 405/488/561/642 nm quad-band dichroic mirror (Chroma, TRF89901v2) and focused at the back-focal plane of the ×100 1.46-numerical-aperture oil objective. The focus is laterally shifted along the back-focal plane to transit from epifluorescence (EPI, 0°) to highly inclined and laminated optical sheet (HiLo, 55.2°) or total internal reflection fluorescence (TIRF, 66.7°) illumination. The fluorescence was collected by the same objective and guided to one of the two pco.edge 4.2 sCMOS cameras through either a 560-nm longpass filter or a dual bandpass filter (bandpass, 490–560 nm; longpass, 640 nm) depending on the sample. The denoise option on the sCMOS camera was used to reduce the noise patterns of the sCMOS camera. Laser intensity used was between 1 and 4 kW cm^–2^. For collecting the EC spectrum, typically ~200 frames were collected with an exposure time of 25 ms.

The datasets shown in Figs. 2 and 3 and Extended Data Figs. 3 and 4 were collected on a Leica Stellaris 8 Falcon FLIM microscope equipped with a resonance scanner, a white light laser and HyD detectors. For the six-colour imaging (Fig. 2), the 488-nm, 561-nm and 638-nm laser lines from the white light laser were used for excitation. A 488/561/638-nm notch filter was placed in front of the white light laser to block any leaked laser light from other spectral regions. Sequential line scanning was performed using the resonance scanner in a bidirectional scan mode at 8 kHz, with sequential detection used to minimize cross-excitation and cross-talk in the emission. The fluorescence was separated using a tunable acousto-optical beamsplitter system, with the bandwidth for green, red and far-red spectral detection set to 495–550 nm, 570–630 nm and 650–720 nm, respectively. For the four-colour imaging shown in Fig. 3, the 561-nm laser line was used for excitation, and the detection bandwidth was set to 570–645 nm. The analogue mode of the HyD detector was used, with the gain set to approximately 30 to prevent pixel saturation. Frame averaging of 6 was used to increase the signal-to-noise ratio, resulting in a frame rate of approximately 200 ms.

For electrochemical fluorescence modulation for colour unmixing, the electrochemical potential was linearly scanned from −0.8 V to 0.15 V and then back to −0.8 V at a rate of 500 mV s⁻^1^. The positive potential limit of 0.15 V was chosen instead of 0.4 V to minimize photobleaching during imaging. Time-lapse series of images were collected during the electrochemical potential scan. To facilitate colour unmixing, reduced image frames at constant potential can be used. For instance, two frames at −0.8 V and −0.15 V can separate two fluorophores, whereas six frames of images at selected potentials as set in Fig. 4 can separate four fluorophores.

Super-resolution STED imaging (Fig. 4, Extended Data Fig. 5 and Supplementary Fig. 3) was performed on the PicoQuant MicroTime 200 STED microscope equipped with a FLIMbee galvanometer scanner (PicoQuant). A 640-nm picosecond pulsed laser (LDH series, PicoQuant) and a 765-nm pulsed STED laser (VISIR-STED, PicoQuant) were set to pulse at 40 MHz to excite and deplete the dyes, respectively. The STED doughnut was achieved by an easy STED approach^33^, where a four-segmented *λ*/2 waveplate with different polarization orientations was inserted underneath a 100× oil-immersion objective (numerical aperture, 1.40; UPlanSApo, Olympus). The refection index of the phase plate causes the STED beam to interfere at the focal centre, forming a focal doughnut, whereas the 640-nm excitation remains unaffected. The STED pulse was electronically delayed by ~200 ps relative to the 640-nm laser to provide the maximum depletion efficiency for the four dyes selected. The STED pulse was widened to ~300 ps to avoid two-photon excitation. The two lasers were reflected to the objective by a 640/760-nm dichroic mirror (Chroma). Samples were imaged and the emitted fluorescence was collected by the same objective and spatially filtered by a 100-µm pinhole at the conjugated focal plane. The pinhole-purified fluorescence was guided and focused onto the single-photon avalanche diodes (SPCM AQRH-14 TR, Excelitas Technologies). A 690/70 bandpass filter (Chroma) was inserted in front of a single-photon avalanche diode detector to clean up the reflected excitation laser, STED laser and stimulated emissions. The cumulative pixel dwell time was 30 µs, and the pixel size was set to 60 nm. To increase the speed of image acquisition, bidirectional scanning was adopted. The phase shift between the forward and reverse line scanning was offline corrected in a custom-written MATLAB_R2023b script, where the peak of the cross-correlation between the adjacent lines was fitted to a Gaussian function and shifts back to the centre with subpixel resolution. To minimize photobleaching, six STED images at the electrochemical potential of −0.7 V, −0.4 V, −0.35 V, −0.3 V, −0.15 V and 0.15 V were taken for the four-colour STED imaging.

All the images collected are drift corrected using a custom-written MATLAB script before unmixing. The linear unmixing algorithm used here is reminiscent of the colour unmixing for spectral imaging, where each pixel in the dye-mixed sample is assumed to contain a mixture of reference dyes. Its electrochemical pattern (**I**_**λ**_) is the total summation of the electrochemical pattern of individual reference dyes (**R**_**iλ**_) weighted by their concentration (**C**_**i**_) as **I**_**λ**_ = ∑_**i**_**C**_**i**_ × **R**_**λ**_, where **i** represents the index of the fluorophore. The best solution of **C**_**i**_ is determined by least-squares fitting with constraints to be non-negative values. The set of **C**_**i**_ that produces the least residual difference from the measured **I**_**λ**_ is regarded as the best fit. The linear unmixing algorithm requires the electrochemical fluorescence modulation pattern of individual reference dyes. These data can be separately collected using a reference-dye-only sample or by selecting a region within the mixed-dye sample that contains only the reference dye. The challenge with using a separate reference fluorophore-only sample lies in the requirement to fully synchronize the electrochemical potential with the microscope image acquisition. In the current study, we obtained all the EC spectra for reference fluorophores by selecting regions within the mixed-dye sample that contained only the reference. To enhance the contrast between different dyes, we generated a colour-coated image by overlaying the sum, standard deviation and the ratio of the maximum to minimum values at each pixel location across the time domain.

Cross-talk analysis: the cross-talk between the electrochemical unmixed images was quantified by Pearson-correlation-coefficient-based co-localization analysis from another study^23^. It is assumed that the imaged structures in the current study are independently spatially distributed. Therefore, zero correlation is expected between the electrochemical unmixed images. If structure A is falsely misidentified in the image of structure B, it would result in a positive correlation between the two images. Conversely, a negative correlation arises when the presence of structure A reduces the signal intensity in the corresponding regions of structure B, resulting in spatial exclusion. The Pearson correlation coefficient *R* is used as a cross-talk factor in the figures to indicate the levels of cross-talk, which is calculated as

$$R=\,\sum \left({{\bf{I}}}_{{\rm{a}}}-{\bar{{\bf{I}}}}_{{\rm{a}}}\right)\left({{\bf{I}}}_{{\rm{b}}}-{\bar{{\bf{I}}}}_{{\rm{b}}}\right)/\sqrt{\sum {\left({{\bf{I}}}_{{\rm{a}}}-{\bar{{\bf{I}}}}_{{\rm{a}}}\right)}^{2}\sum {\left({{\bf{I}}}_{{\rm{b}}}-{\bar{{\bf{I}}}}_{{\rm{b}}}\right)}^{2}},$$

where **I**_a_ and **I**_b_ are Otsu’s threshold-processed images, with the trough value in the intensity histogram subtracted to reduce the background noise. ${\bar{{\bf{I}}}}_{{\rm{a}}}$ and ${\bar{{\bf{I}}}}_{{{\rm{b}}}}$ are the mean values of *I*_a_ and *I*_b_, respectively.

## Online content

Any methods, additional references, Nature Portfolio reporting summaries, source data, extended data, supplementary information, acknowledgements, peer review information; details of author contributions and competing interests; and statements of data and code availability are available at 10.1038/s41566-025-01672-7.

## Supplementary information

Supplementary Information Supplementary Figs. 1–3 and Notes for Supplementary Videos 1–7.

Supplementary Video 1 The *x*–*z* cross-section imaging of ATTO 655-labelled microtubules shows that ATTO 655 exhibits nearly synchronized fluorescence modulation across the entire cell *z* range (~10 µm). The electrochemical potential scan rate is 4 V s^–^^1^ and the exposure time is 20 ms.

Supplementary Video 2 Electrochemical fluorescence modulation of ATTO 655-labelled microtubules over 105 cycles. Potential scan rate is 4 V s^–^^1^; the exposure time is 11 ms.

Supplementary Video 3 Electrochemical fluorescence modulation of six fluorophores in the 488-nm, 561-nm and 642-nm channels. In the imaged HeLa cell, microtubules were labelled with EGFP; vimentin, with Alexa 488; the nucleus, with mCherry; the transferrin receptor, with Alexa 568; actin, with ATTO 655; and paxillin, with STAR 635. Potential scanned at 500 mV s^–1^. Frame time, 200 ms.

Supplementary Video 4 Electrochemical unmixing of ATTO 655-actin and STAR RED-mitochondria in a U-2 OS cell at varying *z* distances. Potential scanned at 500 mV s^–1^. Frame time, 250 ms.

Supplementary Video 5 Electrochemical unmixing of ATTO 655-actin and STAR RED-mitochondria in a U-2 OS cell based on an increasing number of raw image frames.

Supplementary Video 6 Electrochemical fluorescence modulation of four fluorophores in the 561-nm channel in a HeLa cell. The fluorophores are mCherry labelled on H2B, Alexa 555 labelled on actin, Alexa 568 tagged on transferrin and Alexa 594 labelled on mitochondria. Potential scanned at 500 mV s^–1^. Frame time, 200 ms.

Supplementary Video 7 STED images of a U-2 OS cell under various potentials. The cell was co-immunostained with STAR RED-tubulin, ATTO 655-phalloidin, SiR-DNA and STAR 635-paxillin. The applied potentials were set at −0.7 V, −0.4 V, −0.35 V, −0.3 V, −0.15 V and 0.15 V. The STED images were captured at a frame rate of 27 s.

## Source data

Source Data Fig. 1 Raw fluorescence intensity data.

Source Data Fig. 2 Raw fluorescence intensity data and box-plot data.

Source Data Fig. 3 Raw fluorescence intensity data and box-plot data.

Source Data Fig. 4 Raw fluorescence intensity data and box-plot data.

Source Data Extended Data Fig. 1 Raw fluorescence intensity data.

Source Data Extended Data Fig. 2 Summary of the fluorescence intensity data.

Source Data Extended Data Fig. 3 Fluorescence intensity data.

Source Data Extended Data Fig. 4 Fluorescence intensity data and cross-talk data.

Source Data Extended Data Fig. 5 Fluorescence intensity data and box-plot data.

## Data Availability

Source data for this paper are available via Dryad at 10.5061/dryad.cnp5hqcg0. Part of the raw data is not suitable for distribution through public repositories due to the large file size and is available from the corresponding authors upon request. Source data are provided with this paper.

## Code availability

Custom MATLAB (MathWorks) codes used for data processing are available via GitHub at https://github.com/mayuanqing8/Electrochemical-color-unmixing.
