# Supplementary material for: Electrochemical fluorescence modulation enables simultaneous multicolour imaging
Source: Nat Photonics. 2025 May 2;19(7):718–24. doi: 10.1038/s41566-025-01672-7 (PMC12226343; doi:10.1038/s41566-025-01672-7)
Supplement: Supplementary file 1 — Supplementary Figs. 1–3 and Notes for Supplementary Videos 1–7. [file 41566_2025_1672_MOESM1_ESM.pdf]

nature photonics

Supplementary information https://doi.org/10.1038/s41566-025-01672-7

Electrochemical fluorescence modulation enables simultaneous multicolour imaging

In the format provided by the authors and unedited

Table of Contents:

Supplementary Fig.1 EC spectra under varying electrochemical potential scanning rates

Supplementary Fig. 2 Colour unmxing on widefield fluorescence microscopy

Supplementary Fig. 3 Raw confocal and STED microscopy images

Notes for Supplementary Videos

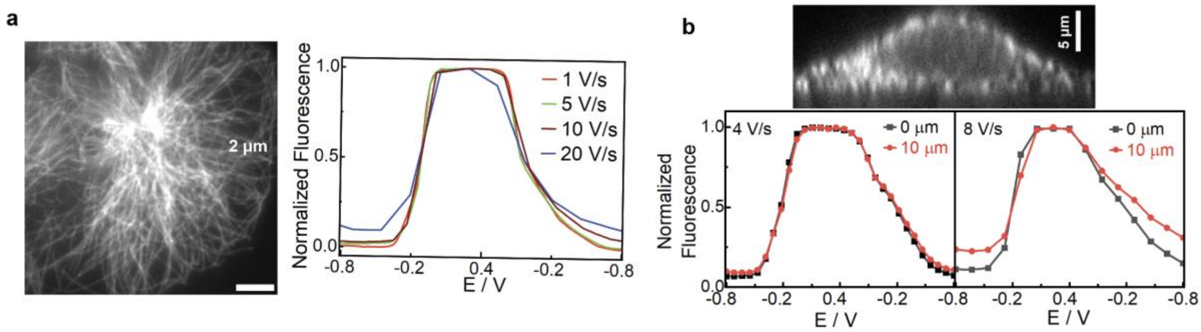

Supplementary Fig. 1. EC spectra for ATTO 655 under varying electrochemical potential scanning rates. a, In the x-y plane imaging near the surface, the EC spectra remain highly consistent up to a potential scanning rate of 10 V s^{-1}, the imaging frame rate was 11 ms. **b,** In the x-z cross-sectional imaging, the EC spectra maintain consistency within a 10 μm range when the potential scanning rate is smaller 4 V s^{-1}, the imaging frame rate was 20 ms. While high imaging speeds are required, a potential scanning rate below 4 V/s is recommended to ensure high consistency in both the x-y and x-z ranges.

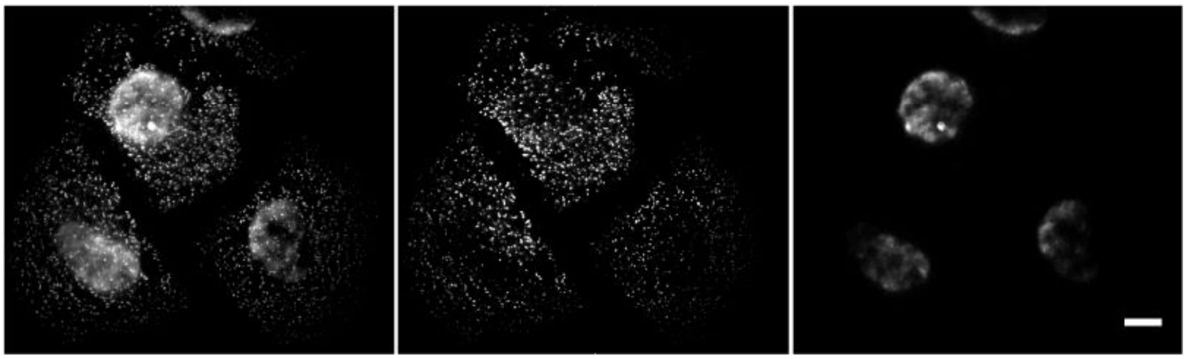

Supplementary Fig. 2. Electrochemical color unmixing demonstration using widefield fluorescence microscopy. Total internal reflection fluorescence (TIRF) images of Hela cells transfected with mCherry-tagged H2B to label the nucleus and labelled with Alexa 568-conjugated transferrin. The left panel shows the raw fluorescence image before color unmixing, where signals from mCherry and Alexa 568 are mixed. The middle and right panels show the separated signals for Alexa 568-labeled transferrin and mCherry-H2B after color unmixing. The scale bar represents 10 μm.

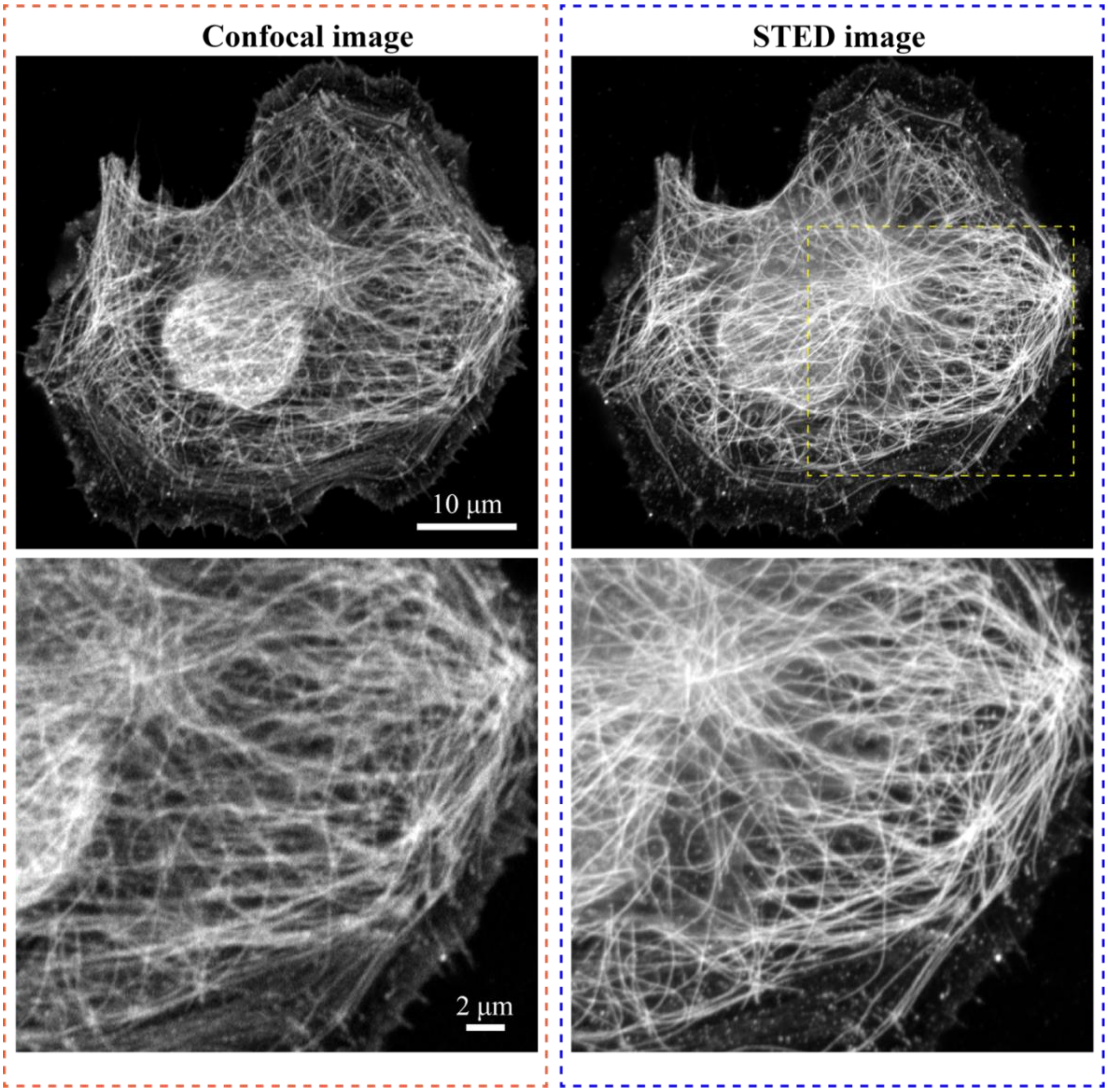

Supplementary Fig. 3. Comparison between raw confocal and STED microscopy images of a U2OS cell with actin stained with ATTO 655, microtubules stained with STAR Red, nuclei labeled with SiR, and paxillin labelled with STAR 635 prior to color unmixing. The zoomed-in region, indicated by the yellow dotted line, shows much finer tubulin structures that can be resolved using STED imaging.

Notes for Supplementary Videos

Supplementary Video 1. ATTO 655 labelled on cellular microtubules exhibits nearly synchronized fluorescence modulation across the whole cell z-range (~10 µm) as the electrochemical potential was scanned from -0.8 V to 0.4 V and back to -0.8 V at a scan rate of 4 V s^{-1}, the frame rate is 20 ms for x-z cross section imaging. The imaging sample is a COS-7 cell on ITO, with microtubules stained using a rabbit anti-alpha tubulin antibody, followed by a secondary ATTO 655-tagged anti-rabbit antibody.

Supplementary Video 2. Electrochemical fluorescence modulation of ATTO 655 labelled on cellular microtubules over 105 cycles. The electrochemical potential was scanned from -0.8 V

to 0.4 V and back at a scan rate of 4 V s^{-1}. The frame rate was 11 ms for x-y plane imaging. Over 105 cycles, a gradual decline in fluorescence intensity is observed due to photobleaching, while the overall modulation pattern remains consistent.

Supplementary Video 3. The fluorescence response of fluorophores in the 488 nm, 561 nm, and 642 nm channels was measured during the linear scanning of the electrochemical potential. For the Hela cell in the movie, microtubules were genetically tagged with EGFP and vimentin stained with Alexa 488; the nucleus (H2B) was genetically tagged with mCherry, and the transferrin receptor stained with Alexa 568; actin was stained with ATTO 655, and paxillin labelled with STAR 635. The potential was scanned from -0.8 V to 0.15 V and then back to -0.8 V at a rate of 500 mV s^{-1}. Images were captured with a frame acquisition time of approximately 200 ms.

Supplementary Video 4. Electrochemical unmixing results for U2OS cells labelled with ATTO 655-actin and STAR Red-mitochondria at various z-distances from the ITO surface. The potential was scanned from -0.8 V to 0.4 V and then back to -0.8 V at a rate of 500 mV s^{-1}. Images were captured with a frame acquisition time of approximately 250 ms.

Supplementary Video 5. Unmixed images of ATTO 655-actin and STAR red-mitochondria in a U2OS cell, showing improved unmixing with an increasing number of frames (2–18 frames) used for electrochemical color unmixing. The potential was scanned from -0.8 V to 0.4 V and then back to -0.8 V at a rate of 500 mV s^{-1}. Images were captured with a frame acquisition time of approximately 250 ms.

Supplementary Video 6. The fluorescence response of four red-emitting fluorophores to a linear scanning electrochemical potential in the 561 nm channel. The fluorophores include mCherry labelled on H2B, Alexa Fluor 555 labelled on actin, Alexa Fluor 568 tagged on transferrin, and Alexa 594 labelled on mitochondria. The potential was scanned from -0.8 V to 0.15 V and then back to -0.8 V at a rate of 500 mV s^{-1}. Images were captured with a frame acquisition time of approximately 200 ms.

Supplementary Video 7. Stack for six STED images of a U2OS cell under various applied potentials. The cell was co-immunostained with STAR Red-tubulin, ATTO 655-phalloidin, SiR-DNA, and STAR 635-paxillin. The applied potentials were set at: -0.7 V, -0.4 V, -0.35 V, -0.3 V, -0.15 V, and 0.15 V, respectively. The STED images were captured with a frame rate of 27 s.
